# Research Progress on the Early Monitoring of Pine Wilt Disease Using Hyperspectral Techniques

**DOI:** 10.3390/s20133729

**Published:** 2020-07-03

**Authors:** Weibin Wu, Zhenbang Zhang, Lijun Zheng, Chongyang Han, Xiaoming Wang, Jian Xu, Xinrong Wang

**Affiliations:** 1 College of Engineering, South China Agricultural University, Guangzhou 510642, China; wuweibin@scau.edu.cn (W.W.); zhenbangzhang@stu.scau.edu.cn (Z.Z.); 201619030305@stu.scau.edu.cn (C.H.); ebianwxm1234@stu.scau.edu.cn (X.W.); xujianaa@stu.scau.edu.cn (J.X.); 2 Division of Citrus Machinery, China Agriculture Research System, Guangzhou 510642, China; 3 Guangdong Province Key Laboratory of Microbial Signals and Disease Control, College of Agriculture, South China Agricultural University, Guangzhou 510642, China; lijunzheng0514@stu.scau.edu.cn

**Keywords:** pine wood nematode, hyperspectral sensor, drone, forest monitoring, pine wilt disease

## Abstract

Pine wilt disease (PWD) caused by pine wood nematode (PWN, *Bursaphelenchus xylophilus*) originated in North America and has since spread to Asia and Europe. PWN is currently a quarantine object in 52 countries. In recent years, pine wilt disease has caused considerable economic losses to the pine forest production industry in China, as it is difficult to control. Thus, one of the key strategies for controlling pine wilt disease is to identify epidemic points as early as possible. The use of hyperspectral cameras mounted on drones is expected to enable PWD monitoring over large areas of forest, and hyperspectral images can reflect different stages of PWD. The trend of applying hyperspectral techniques to the monitoring of pine wilt disease is analyzed, and the corresponding strategies to address the existing technical problems are proposed, such as data collection of early warning stages, needs of using unmanned aerial vehicles (UAVs), and establishment of models after preprocessing.

## 1. Introduction

Pine wilt disease (PWD) is a major quarantine disease that causes the devastating death of *Pinus* species due to the pine wood nematode (PWN) *Bursaphelenchus xylophilus* (Steiner and Bührer) Nickle [1,2,3,4]. Because of its rapid onset and spread in addition to the resulting high mortality, this disease is very difficult to control [5]. Currently, the disease is widely distributed in eight countries, namely, in North America: the United States, Mexico, and Canada; in East Asia: China, Japan, South Korea, and North Korea; and in Europe: Portugal. However, globally, 52 countries have declared *B. xylophilus* as a quarantine pest and PWD as a quarantine disease [2,3,6,7,8,9,10,11,12]. Among the affected countries, China, Japan, and South Korea are the most severely impacted. In 1979, large numbers of pine trees died in Japan, with numbers of dead trees reaching as high as 2.43 million m^3^ [13]. In South Korea, approximately 78.11 km^2^ of pine forest was destroyed in 2005 [14]. Since PWD was first described in the Sun Zhongshan Mausoleum in Nanjing, China, in 1982, the disease has rapidly spread in the tropical and subtropical regions of China in only a few decades. In recent years, PWD has gradually invaded warm temperate zones and exceeded the 10 °C average annual temperature [15].

According to the Chinese Forestry Administration’s No. 4 National Pine Wood Nematode Epidemic Area Announcement in 2019, PWD is widely distributed in 18 provinces [16]. Many trees have been destroyed, leading to large-scale deaths of pine trees, which has severely disrupted the ecological forestry environment and resulted in devastating losses. Because pine trees are environmentally friendly and valuable to the economy, the Chinese government, in addition to numerous others, attaches great importance to this resource, and have strengthened efforts toward the prevention and control of PWD, especially in recent years. Our group has done a lot of work in this field. A wood sampling method was reported in 2010 [17]. It can estimate nematode numbers rapidly in the wood of *Pinus massonina* with only 10 min. In 2011, our group developed a rapid detection of *B. xylophilus* in stored *Monochamus alternatus.* This method can detect nematodes by PCR amplification [18]. One of the key strategies in combatting PWD is the early identification of epidemic areas via large-scale forest monitoring. PWD symptoms can be leveraged for the intelligent monitoring of forest pines [4]. Large-scale forest monitoring of pine wilt disease can be achieved by hyperspectral techniques.

Hyperspectral techniques can be used for object identification and object character analysis. It is widely used in agriculture, oceanography, geology, atmosphere, and environmental sensing [19]. In agriculture, hyperspectral techniques can be used for pest and disease monitoring [20,21,22,23,24,25], biomass estimation [26], and biological stress monitoring [27]. In the field of pest and disease monitoring, it has been applied to many plants, including tomatoes, grapes, and wheat [20,21,22]. Similarly, it can also be applied to the early monitoring of pine wilt disease. However, because hyperspectral techniques are not mature, there are a lot of problems and difficulties that limit its practical applications. Therefore, in order to inspire other research in this area, this review discusses the progress, challenges, and suggestions on the early monitoring of pine wilt disease using hyperspectral techniques.

## 2. PWD Symptoms

PWD is generally transmitted by insect vectors such as *M. alternatus*. When insect vectors feed on pine trees, the pine wood nematode parasite is transferred to pine trees, and PWD caused by the nematode is generally reflected in the canopy of the host plant. PWD is generally divided into three stages on the basis of its symptoms [28]:

(1) The difference between a healthy plant and plant during an early disease stage cannot be observed with the naked eye. The duration of this early stage is mainly affected by pine age and species, environmental temperature, and pine wood nematode virulence. Pine wood nematodes begin to multiply in the xylem of pine trees, blocking the internal ducts of pine trees. Pine trees cannot perform normal water metabolism, and pine resin secretion is reduced along with slight changes in chlorophyll and carotene contents. Clevers’ research showed that the red-edge chlorophyll index is linearly correlated with the canopy chlorophyll content [29]. In addition, Köksal found that the normalized difference vegetation index is strongly associated with the carotene content of leaves [30]. Therefore, the change in chlorophyll and carotene content can be detected by hyperspectral remote sensing.

(2) In the middle stage of the disease, pine tree resin secretion completely stops, and the transpiration effect is weakened. Needles in the crown of the tree gradually become yellowish. Visual differences between healthy and infected pines are evident at this stage. The wilting appearance of the pine during this period differs from that of the healthy pine. PWD causes pine needles to wilt from the inside to the outside.

(3) At the end stage of the disease, needles on the crown of the tree turn brown or reddish brown. The whole plant dies, and the needles do not fall that year.

The spectral characteristics of pines vary according to the type of stress. Different spectral indices can be used to build models to identify PWD and differentiate it from other decline agents, such as bark beetles, defoliating fungi, or even severe droughts [31].

## 3. Traditional PWD Monitoring Technology

### 3.1. PWD Pine Tree Monitoring Methods

**Figure 1:**
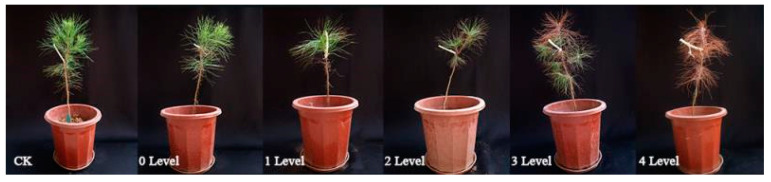
Pine seedlings: healthy plus five different infection levels.

PWD diagnosis depends on human reconnaissance and visual rating (Figure 1), followed by the isolation of nematodes found in diseased wood. Microscopy is used to observe the morphological characteristics of the nematodes and determine whether PWNs are present in the pine tree. Traditional methods of morphological identification require training for recognizing nematodes. Molecular identification is also available but is expensive for large-scale monitoring [32].

In certain harsh conditions that make it difficult to enter a forest, the transmission source cannot be treated in time after pine trees are infected with nematodes, resulting in large-scale damage to the pine forest. In addition, the field diagnosis of PWD based on the analysis of visual symptom is subject to variation due to the different evaluators, and it is easy to draw incorrect conclusions [33,34,35,36]. Many researchers have developed DNA-based methods to rapidly identify PWD using molecular biology methods [37,38,39]. For example, a sampling-staining-PCR detection workflow system was established for the first time by Wang and colleagues [40]. This system can detect pine wood nematodes directly from infected *P. massoniana* within 5 h without requiring nematodes to be isolated from samples. However, this method does not address the problem of allowing pine wood nematode diagnosis before disease symptoms appear (Figure 1).

### 3.2. Pine Wood PWD Monitoring Technology

PWD can be monitored with the “punching and flowing resin” method since this disease leads to a reduction in resin secretion from pine trees. Because this method is not specific to PWD, it was gradually eliminated [41]. On the other hand, PWD can also be diagnosed by monitoring the density of *M*. *alternatus*. *M*. *alternatus* is abundant in pine forests, but this insect does not always transmit the PWN responsible for PWD. In fact, its odds of carrying PWN range from 0 to 60%, and few of these PWNs successfully invade tissue. Therefore, monitoring PWD on the basis of *M. alternatus* is ineffective [42].

Traditional methods for monitoring PWD are time-consuming and labour-intensive. This disease can be diagnosed with certainty by nematode separation and morphology identification. Therefore, an efficient method is urgently needed for the intelligent monitoring of PWD.

## 4. Principles of Monitoring PWD with Hyperspectral Remote Sensing Technology

**Figure 2:**
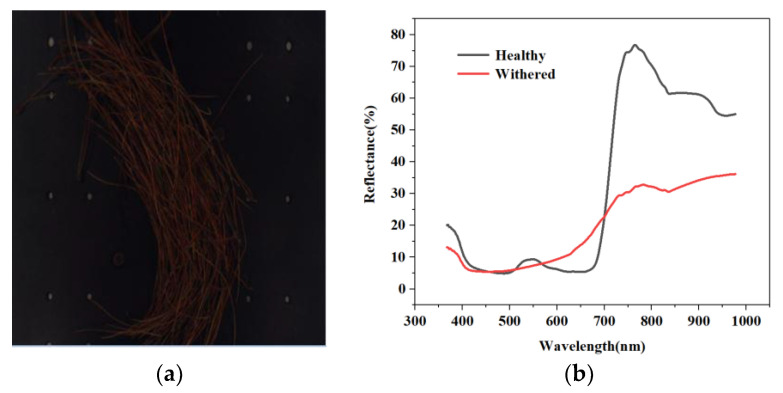
Detection of pine wilt disease using hyperspectral techniques (**a**) Hyperspectral images of diseased pine needles (**b**) Hyperspectral reflectance of healthy and withered masson pine.

The advantage of using hyperspectral remote sensing technology in disease classification is that it can combine image information and spectral information. Image information can be used to analyse surface quality features such as sample size, profiles, and defects. Because spectral absorption varies from one component to another, an image reflects a certain feature at characterised bands, and spectral information can fully reflect a discrepancy in the physical structure and chemical composition of a sample [43]. These characteristics determine the unique advantages of spectral imaging technology in testing the internal and external quality of farm products. Spectral imaging technology makes full use of the absorption or radiation characteristics of a substance in different electromagnetic spectra. One-dimensional spectral information is added on the basis of ordinary two-dimensional space imaging. Due to differences in the composition of various substances, differences also exist between their corresponding spectra such that the spectrum of the target can be used in identification and classification. To achieve monitoring, plant diseases are identified through various parameters, such as morphological changes, temperature changes, transpiration rates, and volatile organic compound release by pathogen-infected plants [44]. The delicate process of host–pathogen interactions also affects the optical characteristics of plants to a certain extent [45]. Data about these changes can be obtained by sensors. Hyperspectral sensors offer the most abundant data information. This technology can supply sufficient evidence, demonstrating its feasibility for the early detection of plant diseases. With the development of hyperspectral technology in recent years, increasing numbers of researchers have applied it to plants, including for plant-nutrition monitoring [46], the automatic classification and monitoring of plant diseases [47,48], and their early detection [45], as well as to obtain digital phenotypes for disease-resistant plant breeding [49,50]. For example, pine wilt disease was diagnosed using hyperspectral methods by Kim’s group. In their research, significant differences were observed in vegetation indices such as the normalized difference vegetation Index (NDVI), the green normalized difference vegetation index (GNDVI), the plant senescence reflectance index (PSRI), and the pigment specific normalized difference (PSND), beginning on 20 August 2012 [51]. Our group study the hyperspectral characteristic of healthy and withered masson pine, and Figure 2 shows the research results. If a plant is infected with PWD, the green needles of the pine trees begin to change colour. The spectral reflectance of pines varies over the different degrees of infection. Therefore, disease level can be evaluated on the basis of spectral reflectance.

Spectral imaging technology can collect many narrow and continuous hyperspectral reflectance datasets in the ultraviolet, visible, near-infrared, and mid-infrared regions of the electromagnetic band, providing complete and continuous spectral curves for each pixel. Imaging spectrometers can be divided according to their resolution, as follows [52].

Multispectral imagers (MSIs): the obtained target has a wavelength band between 320 and 1300 nm, and the spectral resolution is generally close to 100 nm; this technology is mainly used in zone classification [53].

Hyperspectral imagers (HSIs): the obtained target band is between 350 and 2500 nm, and the spectral resolution is approximately 10 nm; this technology is widely used in various fields, such as food safety, quality, and biomedical agricultural product testing [54].

Ultrahyperspectral imagers (USIs): the obtained target band is between 1000 and 10,000 nm, and the spectral resolution is below 1 nm; this technology is commonly used when fine detection is required, such as in the case of atmospheric particle detection [55].

## 5. Hyperspectral Technology in Monitoring of PWD in Forests

### 5.1. Forest Surveillance of Dead Pine Wilt Disease

As early as 1994, Wu and colleagues, who monitored the Chinese pine caterpillar and pine wood nematode (*Bursaphelenchus xylophilus*), began to combine high-quality super VHS (video home system) camera systems with global positioning system (GPS) navigation, image processing, and geographic information systems. The captured image data could be converted into digital data for further analysis [56]. Methods based on the Korea Mahalanobis distance (MD) and maximum likelihood were used to classify TM (thematic mapper) and IKONOS images in 1998. In addition, the GIS (geographic information system) spatial analysis function was combined with ground survey data information to predict disasters [57]. 

The Mahalanobis distance (MD) is defined as follows [58]:

(1) $$D_{M}\left(x\right)=\sqrt{{\left(x-\mu \right)}^{T}S^{-1}\left(x-\mu \right)}$$

where *D_M_* is the Mahalanobis distance between test samples and reference samples, *µ* is the mean vector of the reference samples, and *S* is the covariance estimate of the reference samples.

Maximum likelihood is determined as follows [59]:

*P* = (*x*, *x*_2_, *x*_3_, …, *x*_n_) = *f*_D_(*x*_1_, *x*_2_, …, *x*_n_∣*θ*) *lik*(*θ*) = *f*_D_(*x*, *x*_2_, …, *x*_n_∣*θ*) (2)

**Figure 3:**
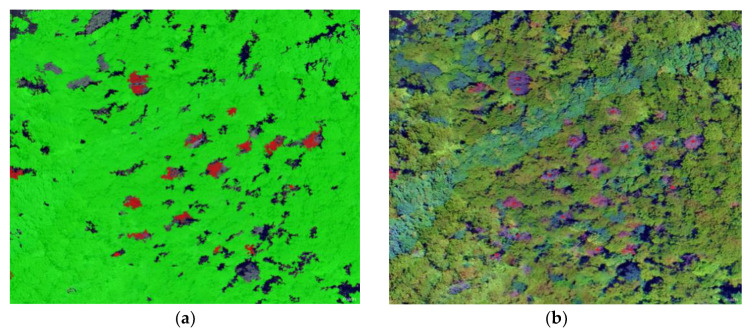
Pine forest photographed by unmanned aerial vehicle (UAV). PWD: pine wilt disease. (**a**) Hyperspectral image of PWD; (**b**) Digital image of PWD.

Unmanned aerial vehicles (UAVs) can carry sensors to collect data in inaccessible mountains and obtain higher-resolution image data via software stitching. Figure 3 shows pine forest photographed by unmanned aerial vehicle. Huang and colleagues used small, fixed-wing drones to monitor an epidemic area with a total area of 145.29 km^2^ and automatically identified 1486 dead pine trees. The distribution maps and coordinate-point locations of these dead trees were also obtained. The monitoring accuracy exceeded 80%, and the point accuracy reached 2–3 m [60]. Low-cost, small UAV high-resolution images were used by Li and colleagues to locate a single dead tree with an accuracy of 83%, which offered technical support for the quick identification of suspected dead trees and the determination of the outbreak area [61]. The hue saturation value (HSV) threshold method was used by Tao and colleagues to extract discoloured pine trees with an accuracy of 60–65%, which improved the efficiency of manual interpretation. This method is suitable for the late monitoring of dead pine trees, and lends theoretical and methodological support for monitoring discoloured pine trees on the basis of drone images [62]. The above studies are based on the RGB three-channel sensor in canopy size. However, the visual portion of the electromagnetic spectrum contains only the three bands of red, green, and blue [63]. Due to its high throughput, this sensor is widely used in the field of remote sensing detection, but it also reduces the analysis accuracy. Therefore, it is important to increase the resolution for the early monitoring of PWD.

### 5.2. Forest Monitoring for Early PWD Detection

The early monitoring of PWD has always been a topic of concern. In 2017, a novel method that combined airborne laser scanning with high-resolution spaceborne images was used to detect pine trees affected by PWD, and a support vector machine was applied by Takenaka’s team to classify different degrees of pine disease [64]. Du and others used a portable ASD (analytica spectra devices) FieldSpec full range field spectrometer with a band value between 350 and 2500 nm to collect reflection spectra data of pine needles in the field [65]. Kim’s group used a GER 3700 spectrometer to obtain reflections from infected and uninfected trees [31]. A FieldSpec4 (400–1100 nm) field spectrum analyser was used by Zhang’s group to identify needles with different levels of damage in 70 sampling areas [66]. Wang et al. hyperspectrally recorded black pine older than 20 years-initially healthy-from the date of pine nematode inoculation until their death [67]. Ma and colleagues used the ASD FieldSpec3 spectrometer to measure the hyperspectral reflectance of pine trees in three different areas [68]. Huang et al. used a back-mounted ASD FieldSpec Pro FR field spectroradiometer to measure the needle leaf spectrum in a darkroom [69]. The above studies required that needles be removed from the field to a stable indoor environment to measure their spectral reflectance or, alternatively, the selection of a relatively stable environment to conduct this measurement in the field. The sensor used in the device had a high resolution (350–2500 nm, containing hundreds of bands) and obtained information in the three dimensions of X, Y, and spectrum. However, this needle study was limited to the laboratory, a relatively stable condition, providing data that can support the early detection of PWD in the field. Yet many pine forests are located in places that are difficult to reach, and the early detection of PWD must be performed in the field, which poses many challenges for data collection.

## 6. Drones Equipped with Hyperspectral Sensors to Monitor Forest PWD

Many scientific methods that obtain good results under laboratory conditions often perform poorly in practical applications. In the study of PWD, when it comes to conducting field measurements, researchers can only measure forests that they can reach and test under relatively stable measurement conditions. This situation presents a notable challenge that limits model application in the field. However, solutions have emerged with scientific advances. The combination of drones and hyperspectral technology in precision agriculture has solved many problems in agricultural production. With the miniaturisation of GPS receivers, inertial navigation systems, computers, and remote sensing sensors, increasing numbers of researchers have begun to use UAVs to build data collection platforms [70]. Drones have many advantages that make them suitable for use in forests. For example, drones offer the advantages of low consumables, low operating costs, and high-intensity data collection [71]. Drones can carry several different types of optical sensors that can be assembled on the basis of the investigated parameters and operated in a certain amount of time. This method allows users to optimise flight time to avoid inconsistent light intensity caused by clouds [72], and data can be accurately collected when a pest or wildfire outbreak occurs [71,73]. Finally, drones can collect data at high spatial resolution, which is a key aspect of the early monitoring of PWD. Hence, the advantages of drones are evident. However, because of their small size, the flight endurance time of drones is low, which limits their ability to obtain data over a large area [74]. Payload capacity limits the ability to carry airborne sensors, especially technologies that are not sufficiently miniaturised. In addition, the size and shape of the fuselage influences the installation of sensing equipment [70,71,72]. Additionally, it might take a long time to set up a UAV system, which includes the selection process for aircraft and sensors as well as processes for their installation and adaptation. Finally, it is necessary to conduct test flights in the measured environment.

In the selection of drones, eBee-type microdrones are a type of light fixed-wing drone that can operate completely autonomously, and require almost no driving skills [75]. An eight-rotor remote control drone (Cinestar-8 MK Heavy Lift, Freefly Systems, Redmond, WA, USA) was used in more accurate fixed-point acquisition by Severtson and others [76]. The multirotor design of this drone supplies stable acquisition space. Dash’s group used coaxial four-wing drones to collect multispectral images for the monitoring of leaf discolouration caused by simulated forest disease outbreaks [77]. Sandino used a six-rotor DJI S800 EVO UAV equipped with a Headwall Nano-Hyperspec® hyperspectral camera to collect data that were used to study the harm caused by myrtle rust to paper tea grown near New South Wales, Australia [78].

## 7. Prospects of PWD Monitoring Using Hyperspectral Technology

### 7.1. Hyperspectral Technology in PWD Forest Monitoring

Three different types of sensors are used to study hyperspectral features: RGB, multispectral (MS), and hyperspectral (HS) sensors [79]. A hyperspectral sensor contains hundreds of bands in the electromagnetic region of 350–2500 nm, with only a few nanometres in each band; thus, consistent and accurate changes in spectral characteristics can be displayed on a spectrogram. Because of the objectivity, continuity, and accuracy of hyperspectral imaging technology, it can be used in automated systems [34]_._ Therefore, its combination with drone technology allows the possibility of monitoring areas that are out of human reach.

### 7.2. Hyperspectral Data Acquisition

Hyperspectral sensors can be used to acquire hyperspectral data, and they are divided into two main types: nonimaging [80,81] and imaging sensors. Nonimaging hyperspectral sensors cannot obtain information on spatial dimensions, and can only measure the average spectral reflectance of the region of interest. Imaging sensors are further divided into three types: push, broom, and filter-based sensors [64,82].

Hyperspectral imaging sensors must capture one-dimensional spectral target information while capturing two-dimensional spatial image information. However, the array of illumination sensors can only detect information in two dimensions. Stirring broom and push-type scanners eliminate this difference by simultaneously capturing spectral information of lines or points on the measured object [81]. The object of interest is scanned by moving or rotating it to form a hyperspectral image. The advantage of these two hyperspectral sensors is that they can supply fine spectral resolution and collect more effective information. On the other hand, these sensors also have certain disadvantages. Due to the speed limitation of target movement or rotation, and the different size of the collection area, it takes a long time to collect images, which limits applications of this approach in agriculture. Filter-based hyperspectral sensors require filter changes during the image acquisition process. The filter allows only specific bands to pass [83]. The image acquisition speed of this kind of sensor is obviously faster than that of the other two types of imaging sensors, but images obtained at different times are inconsistent. This type of camera is known as a fast camera, and if it can simultaneously captures all the bands, the entire image cube is recorded without the scanning process within the same exposure time [84]. Recently, hyperspectral/multispectral snapshot sensors have been introduced in fast cameras. These units use the same mosaic principle as ordinary RGB cameras, and offer faster image acquisition speeds than those of the original hyperspectral imaging sensors.

On the other hand, if bands or band packets are sequentially or asynchronously recorded by different cameras, image cubes must be created by band coregistration. These camera systems are known as image frame cameras [85]. Fast cameras have significantly faster image acquisition speeds than those of image frame cameras, but faster speed also results in lower hyperspectral image spatial resolution. However, the fast recording time and the continuous creation of hyperspectral image sequences make the fast camera highly suitable for early detection of pine wilt disease in selected special environments. These nonintrusive sensors can complete image evaluation and analysis within a short time and have high image spatial resolution [86]. The combination of fast cameras and drones can collect a high amount of data and improve the efficiency of information acquisition [87].

### 7.3. Hyperspectral Image Acquisition Environment

Hyperspectral images of pine wood nematode disease are currently obtained in the laboratory or in the field under relatively stable conditions, but research objects are mostly located in high mountains, steep roads, and dense forest areas, where ground investigation makes timely detection difficult, and environmental conditions are quite complicated.

Image acquisition in the field is affected by many factors, and the most important factor is light intensity [88]. Studies have shown that sunlight is an excellent light source for hyperspectral imaging, and covers a wide range of wavelengths. However, the main problem is that the light intensity of sunlight cannot be controlled. Usually, clouds cover the sun to varying degrees, causing uncertainty in light intensity. A change in light intensity during the time between measurement of the reference template and the sample can lead to inaccurate measurements [89,90]. Light intensity is affected, and the angle of incident light changes at each moment. The angle between incident light, plant, and hyperspectral sensor has a certain effect on the spectral image acquisition of the sample plant [90,91,92,93]. This inaccurate information is gradually enlarged in subsequent information processing, which makes it difficult to obtain real information. Therefore, it is challenging to select light sources in the laboratory. Common light sources have the disadvantage of thermal changes. Generally, this problem can be solved by preheating [94]. To supply sufficient lighting conditions for hyperspectral imaging, many companies and researchers have offered multiple solutions through the implementation of design modifications. The light source commonly used in these solutions is a tungsten halide lamp with different improvements [93,95], and the emitted light is in a spectrum of 350–2500 nm [96]. Nagasubramanian and others selected two 70 W quartz–tungsten halide lamps to supply stable illumination in the study of soybean anthracnose [97]. The lamp was aimed at the sample stage at a 45° angle and at a distance of 54 cm. After data preprocessing, a 3D (3 dimensions), deep convolutional neural network (DCNN) model was used in classification. The model achieved a classification accuracy of 95.73%. In 2015, light-emitting diodes (LEDs) with an emission peak at 470 nm were used by Mahlein and colleagues to establish an illumination array. This array could improve the sensitivity of a hyperspectral imager in the blue spectrum [98].

### 7.4. Hyperspectral Data Analysis

#### 7.4.1. Data Processing

After hyperspectral data were set, the white and black frames were acquired. In the laboratory, with the exception of the computer, all other equipment was placed in a black box to eliminate the interference of external light. The white frame was obtained by measuring white reference objects with reflectance close to 100%, such as barium sulfate, magnesium oxide, and white reference tiles. Black frames were obtained by closing the camera cover to obtain 0% reflectivity [99,100]. Black and white frames can be collected in the field by similar means, and a unified standard hyperspectral image is obtained after calibration. Even a hyperspectral image obtained in the laboratory still contains a large amount of noise, and a large signal-to-noise ratio affects the application of the data. To solve this problem, many studies have measured the sample multiple times during the data measurement stage [101], and used the measurements to reduce the effect of noise. To solve this problem in the data analysis stage, researchers preprocess the image via such methods as Savitzky–Golay (SG) smoothing, standard normalisation (SNV), de-enveloping (CR), logarithmic inverse (LR), normalisation (NDV), and first and second derivatives [92,102,103,104]. The preprocessing of hyperspectral images improves the signal-to-noise ratio of the data, and makes useful information more prominent in the image. In addition, reducing the dimension and denoising the hyperspectral images are more conducive to building accurate models. However, the choice between the preprocessing and machine learning methods has an impact on model construction. Liu and others tested a variety of preprocessing techniques, and used two different machine learning methods. The preprocessing results via SG smoothing combined with partial least-squares regression (PLS) were considered the best, which predicted best for the content of N and P in citrus leaves [102].

#### 7.4.2. Machine Learning Methods Used in PWN Research

**Figure 4:**
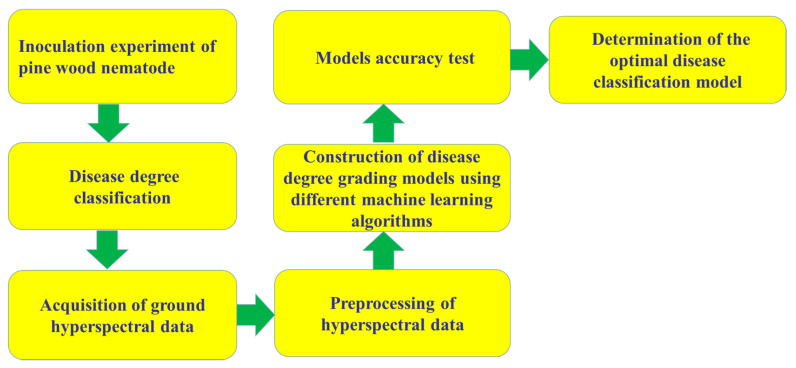
Construction of PWN grading models. PWNL: pine wood nematode, *Bursaphelenchus xylophilus*.

In recent years, the application of hyperspectral technology has ranged from the remote sensing of the Earth’s surface to the identification of all aspects of microbial diseases in plant pathology [105], leading to an increase in the number of different data analysis methods that have been developed. This section describes the machine learning methods used in PWN research. Figure 4 shows the construction process of PWN grading models. Zhang used a spectral angle mapper (SAM) to classify hyperspectral images obtained in field remote sensing research on nematode disease [106]. Spectral angle mappers can reduce the impact of light and terrain, and the classification threshold is lower. The obtained results are more authentic [107,108], and the lower the classification threshold of the SAM, the higher the accuracy. In this study, when the system threshold was set to 0.1, the nematode thresholds in the late and middle stages were 0.06 and 0.01, respectively, and high reliability was obtained in large-scale remote sensing. Lu performed artificial neural network (ANN) research on the prediction of PWD [109]. Artificial neural network regression is a method that uses neural networks to propagate between layers, simulating human brain receiver and message-processing activity [110,111]. ANN is also a learning classification method based on large samples. However, with this method, it is easy to overlearn and reduce the generalisation ability due to the influence of network structure and sample complexity. The most important parameter in the neural network regression model is the number of neurons [112]. The application of ANN is highly extensive, involving all aspects of scientific production [113]. Six stages were adopted in this research to differentiate healthy from diseased pines: healthy, infected, early stage of disease, middle stage of disease, serious stage of disease, and terminal stage of disease [109,114]. Three neural networks were used in the analysis of these six stages. The average accuracy rates of prediction were 64.0% for the BP (back propagation) neural network [115], 72.0% for radial basis neural network (RBF) [116], and 72.3% for the Elman neural network (REN) [117]. Liu of Nanjing Forestry University used a variety of machine learning methods to analyse data in the study of pine physiological parameters under the stress of pine wood nematodes [118]; these methods included support vector machine (SVM) [119], random forest (RF) [120], and the BP neural network. For the analysis by these methods, the *R*^2^ value of the chlorophyll content estimation model of high-intensity *Pinus* was 0.8234 using SVM, 0.7214 using RF, and 0.9022 using the BP neural network. Hence, artificial neural networks showed the best prediction performance.

### 7.5. Challenges and Countermeasures in PWD Monitoring with Spectral Technology

#### 7.5.1. Challenges

(1) Problems with data acquisition equipment. Currently, more effective early detection is needed, and early warnings that can be observed before PWD are required. Regular inspections are required, but humans cannot do this work. The low endurance of machines and the low resolution of their sensors are not sufficient for solving this problem. However, with the introduction of more compact high-resolution sensors, this problem can be solved. Data collected by a high-resolution drone can be verified and classified in the original model to obtain an early warning, or the drone can quickly obtain its own analysis results for classification and marking in a self-contained microcomputer.

(2) Data acquisition environment problems. Sunlight, wind speed, humidity, temperature, and other factors can cause changes in the hyperspectral image of the sample. When using an UAV in the field for cruise warning activities, it is necessary to choose conditions similar to those in the laboratory. The goal is to make it easier to translate scientific results into PWD field monitoring.

(3) Construction of a data analysis prediction model. This is a substantial problem in the early monitoring of PWD and the hyperspectral monitoring of many diseases. Model construction is carried out with the support of data, which means that additional data are required for analysis, such that the model can obtain higher accuracy. Stable data collection must be performed under laboratory conditions, and data collected in the field need additional data corrections, such as for light radiation and ground objects.

(4) Time gap in early detection. There may exist a short time gap between detecting time and PWD revealed time when we try early detection. It can be one- or two-week difference, so adequate isolation or cutting can be the management option at the current stage.

#### 7.5.2. Conclusions and Suggestions

In the current research, multispectral imagers in combination with UAV technology have mostly been used to directly acquire spectral images in the field. On the one hand, multispectral imagers have been miniaturised, and their weight and volume are compatible for their use with drones. On the other hand, many studies are still in the initial stages, with the concept of digital phenotype still being tested, and fewer studies are available regarding the spectral characteristics of the early symptoms of the disease. The early detection of disease can provide strong evidence for the control of the source of transmission, but internal changes of the host during the incubation period or in the early stage of the disease are indistinguishable to the naked eye; the high resolution of hyperspectral imagers can offer more fine-grained data for analysis. Currently, the portability of hyperspectral imagers offers greater possibilities for effective application of this technology. In terms of stability, the distance between the camera and target object severely affects data stability. Distance control is particularly critical for dynamic acquisition in the field. Distance affects the exposure of natural light and the camera’s range of acquisition. Introducing a wide-angle or fisheye lens to increase the acquisition range and reduce the number of acquisitions could greatly reduce image instability. It should be noted that, in order to reduce the influence of the external environment and ensure the accuracy of experimental results, the hyperspectral camera should be used at noon on sunny day. Owing to technical limitations, the signal-to-noise ratio of the obtained spectral data is too low. The mainstream method uses a variety of preprocessing methods and useful data learning models to analyse data. After the pine is infected with PWD, the pine needles begin to turn yellow, and the difference of hyperspectral reflectivity becomes more obvious. Therefore, it is much easier to monitor pine wilt disease using hyperspectral images, but the prediction models need to be retrained using samples infected with PWD. With the continued development of science, the technology of hyperspectral monitoring has the potential to make greater contributions to the monitoring of PWD.

## References

[1] Kim N., Jeon H.W., Mannaa M., Jeong S., Kim J., Lee C., Park A.R., Kim J., Seo Y. (2019). Induction of resistance against pine wilt disease caused by *Bursaphelenchus xylophilus* using selected pine endophytic bacteria. Plant Pathol..

[2] Mota M.M., Vieira P. (2008). Pine Wilt Disease: A Worldwide Threat to Forest Ecosystem.

[3] Abelleira A., Picoaga A., Mansilla J.P., Aguin O. (2011). Detection of *Bursaphelenchus xylophilus*, causal agent of pine wilt disease on Pinus pinaster in Northwest Spain. Plant Dis..

[4] Futai K. (2013). Pine Wood Nematode, *Bursaphelenchus xylophilus*. Annu. Rev. Phytopathol..

[5] He L.X., Ji J., Qiu X.W. (2014). Occurrence and control measures of pine wood nematode disease in the world. J. For. Eng..

[6] Esuer M., Arias M., Bello A. (2004). Occurrence of the genus *Bursaphelenchus* Fuchs, 1937 (Nematoda: Aphelenchida) in the Spanish conifer forests. Nematology.

[7] Futai K. (2003). Role of asymptomatic carrier trees in epidemic spread of pine wilt disease. J. For. Res..

[8] Jones J.T., Moens M., Mota M. (2008). *Bursaphelenchus xylophilus*: Opportunities in comparative genomics and molecular host-parasite interactions. Mol. Plant Pathol..

[9] Proenca D., Romeu F., Santos C.V., Andre L., Luis F., Abrantes L.M., Morais P.V. (2010). Diversity of bacteria associated with *Bursaphelenchus xylophilus* and other nematodes isolated from *Pinus pinaster* trees with pine wilt disease. PLoS ONE.

[10] Fonseca L., Cardoso J., Lopes A. (2012). The pinewood nematode, *Bursaphelenchus xylophsilus*, in Madeira Island. Helminthologia.

[11] Erevková A., Mota M., Vieira P. (2014). *Bursaphelenchus xylophilus* (Steiner & Bührer, 1934) Nickle 1970—Pinewood nematode: A threat to European forests. For. J..

[12] Diogo N.P., Gregor G., Paula V.M. (2017). Understanding pine wilt disease: Roles of the pine endophytic bacteria and of the bacteria carried by the disease—Causing pinewood nematode. Microbiol. Open.

[13] Kishi Y. (1995). The Pine Wood Nematode and the Japanese Pine Sawyer.

[14] Shin H., Lee H., Woo K.S., Noh E.W., Koo Y.B., Lee K.J. (2009). Identification of genes up regulated by pine wood nematode inoculation in Japanese red pine. Tree Physiol..

[15] Li Y., Zhang X. (2018). Analysis on the trend of invasion and expansion of *Bursaphelenchus xylophilus*. Pest Dis..

[16] Announcement of the State Forestry and Grassland Administration (2019 No. 4) (Pinewood Nematode Epidemic Area in 2019). http://www.forestry.gov.cn/.

[17] Wang X.R., Kong X.C., Jia W.H., Zhu X.W., Ren L.L., Moto M.M. (2010). A rapid staining-assisted wood sampling method for PCR-based detection of pine wood nematode *Bursaphelenchus xylophilus* in *Pinus massoniana* wood tissue. For. Pathol..

[18] Wang X.R., Zhu X.W., Kong X.C., Moto M.M. (2011). A rapid detection of the pinewood nematode, *Bursaphelenchus xylophilus* in stored *Monochamus alternatus* by rDNA amplification. J. Appl. Entomol..

[19] Abedin M.N., Refaat T.F., Bhat I.B. (2004). Progress of Multicolor Single Detector to Detector Array Development for Remote Sensing. Proc. Spie Int. Soc. Opt. Eng..

[20] Krezhova D., DiKova B., Maneva S. (2014). Ground based hyperspectral remote sensing for disease detection of tobacco plants. Bulg. J. Agric. Sci..

[21] Vanegas F., Bratanov D., Powell K., Weiss J., Gonzalez F. (2018). A Novel Methodology for Improving Plant Pest Surveillance in Vineyards and Crops Using UAV-Based Hyperspectral and Spatial Data. Sensors.

[22] Mewes T., Franke J., Menz G. (2011). Spectral requirements on airborne hyperspectral remote sensing data for wheat disease detection. Springer Sci. Bus. Media.

[23] Abdulridha J., Ampatzidis. Y., Kakarla S.C., Roberts P. (2019). Detection of target spot and bacterial spot diseases in tomato using UAV-based and benchtop-based hyperspectral imaging techniques. Precis. Agric..

[24] Shadrin D., Pukalchik M., Uryasheva A., Tsykunov E., Yashin G., Rodichenko N., Tsetserukou D. (2004). Hyper-spectral NIR and MIR data and optimal wavebands for detection of apple tree diseases. arXiv.

[25] Gu Q., Sheng L., Zhang T., Lu Y., Zhang Z., Zheng K., Hu H., Zhou H. (2019). Early detection of tomato spotted wilt virus infection in tobacco using the hyperspectral imaging technique and machine learning algorithms. Comput. Electron. Agric..

[26] Smith M.L., Ollinger S.V., Martin M.E., Aber J.D., Hallett R.A., Goodale C.L. (2002). Direct estimation of aboveground forest productivity through hyperspectral remote sensing of canopy nitrogen. Ecol. Appl..

[27] Nigam R., Kot R., Sandhu S.S., Bhattacharya1 B.K., Chandi R.S., Singh M., Singh J., Manjunath K.R. (2016). Ground Based Hyperspectral Remote Sensing to Discriminate Biotic Stress in Cotton Crop. Proc. Spie.

[28] Santos C.S.S.D., Vasconcelos M.W.D. (2012). Identification of genes differentially expressed in *Pinus pinaster* and *Pinus pinea* after infection with the pine wood nematode. Eur. J. Plant. Pathol..

[29] Clevers J.G.P.W., Koolstra L. (2012). Using Hyperspectral Remote Sensing Data for Retrieving Canopy Chlorophyll and Nitrogen Content. IEEE J. Sel. Top. Appl. Earth Obs. Remote Sens..

[30] Koksal E.S., Üstun H., Özcan H., Gunturk A. (2010). Estimating water stressed dwarf green bean pigment concentration through hyperspectral indices. Pak. J. Bot..

[31] Kim S.R., Kim E.S., Nam Y., Choi W.I., Kim C.W. (2015). Distribution characteristics analysis of pine wilt disease using time series hyperspectral aerial imagery. Korean J. Remote Sens..

[32] Moens M., Subbotin S., Maafi Z.T. (2003). Molecular identification of cyst-forming nematodes (Heteroderidae) from Iran and a phylogeny based on ITS-rDNA sequences. Nematology.

[33] Akintayo A., Tylka G.L., Singh A.K., Ganapathysubramanian B., Singh A., Sarkar S. (2018). A deep learning framework to discern and count microscopic nematode eggs. Sci. Rep..

[34] Bock C.H., Poole G.H., Parker P.E., Gottwald T.R. (2010). Plant disease severity estimated visually, by digital photography and image analysis, and by hyperspectral imaging. Crit. Rev. Plant Sci..

[35] Naik H.S., Zhang J., Lofquist A., Assefa T., Sarkar S., Ackerman D., Singh A.K., Ganapathysubramanian B. (2017). A real-time phenotyping framework using machine learning for plant stress severity rating in soybean. Plant Methods.

[36] Zhang J., Naik H.S., Assefa T., Sarkar S., Chowda Reddy R.V., Singh A., Ganapathysubramanian B., Singh A.K. (2017). Computer vision and machine learning for robust phenotyping in genome-wide studies. Sci. Rep..

[37] Harmey J.H., Harmey M.A. (1993). Detection and identification of *Bursaphelenchus* species with DNA fingerprinting and polymerase chain reaction. J. Nematol..

[38] Abad P., Mota M., Vieira P. (2004). Satellite DNA used as a species specific probe for identification of the pine wood nematode *Bursaphelenchus xylophilus*. EPPO Bull..

[39] Akeuchi Y., Kanzaki N., Futai K. (2005). A nested PCR-based method for detecting the pine wood nematode, *Bursaphelenchus xylophilus*, from pine wood. Nematology.

[40] Wang X., Zhu X., Hu Y., Huang H., Kong X., Jia W. (2009). A PCR-Based Method for Detecting *Bursaphelenchus xylophilus* from *Monochamus alternatus*. Sci. Silvae Sin..

[41] Kenichi Y., Takuma T., Natsumi K., Komatsu M., Levia D.F., Kabeya D., Tobita H., Kitao M., Ishida A. (2018). Pine wilt disease causes cavitation around the resin canals and irrecoverable xylem conduit dysfunction. J. Exp. Bot..

[42] Kuroda K. (2008). Physiological incidences related to symptom development and wilting mechanism. Pine Wilt Disease.

[43] Goet A.F.H., Vane G., Solomon J.E., Rock B.N. (1985). Imaging spectrometry for earth remote sensing. Science.

[44] Fang Y., Ramasamy R. (2015). Current and prospective methods for plant disease detection. Biosensors.

[45] Mahlein A.K., Kuska M.T., Behmann J., Polder G., Walter A. (2018). Hyperspectral sensors and imaging technologies in phytopathology: State of the Art. Annu. Rev. Phytopathol..

[46] Meggio F., Zarco-Tejada P.J., Núñez L.C., Sepulcre-Cantób G., Gonzálezc M.R., Martin P. (2010). Grape quality assessment in vineyards affected by iron deficiency chlorosis using narrow-band physiological remote sensing indices. Remote Sens. Environ..

[47] Barbedo J.G.A. (2013). Digital image processing techniques for detecting, quantifying and classifying plant diseases. Springerplus.

[48] Barbedo J.G.A. (2016). A review on the main challenges in automatic plant disease identification based on visible range images. Biosyst. Eng..

[49] Fiorani F., Schurr U. (2013). Future scenarios for plant phenotyping. Annu. Rev. Plant Biol..

[50] Araus J.L., Kefauver S.C., Zaman-Allah M., Olsen M.S., Cairns J.E. (2018). Translating high-throughput phenotyping into genetic gain. Trends Plant Sci..

[51] Kim S.R., Lee W.K., Lim C.H., Kim M., Kafatos M.C., Lee S.H., Lee S.S. (2018). Hyperspectral analysis of pine wilt disease to determine an optimal detection index. Forests.

[52] Wendel A., Underwood J. (2017). Illumination compensation in ground based hyperspectral imaging. ISPRS J. Photogramm. Remote Sens..

[53] Shamsoddini A., Trinder J.C., Turner R. (2013). Pine plantation structure mapping using worldview-2 multispectral image. Int. J. Remote Sens..

[54] Lorente D., Aleixos N., Gómez-Sanchis J., Cubero S., García-Navarrete O.L., Blasco J. (2012). Recent advances and applications of hyperspectral imaging for fruit and vegetable quality assessment. Neuroimage.

[55] Prieto A., Bellas F., Lopez-Pena F., Duro R.J. (2008). Automatic preprocessing and classification system for high resolution ultra and hyperspectral images. Computational Intelligence for Remote Sensing.

[56] Wu J., Ma X.M., Li Z.Q., Gao S. (1994). An evaluation of airborne videography for detecting and monitorIng forest insect and disease. Rorest Res..

[57] Kim J.B., Jo M.H., Oh J.S., Lee K.J., Park S.J. Extraction method of dam-aged area by pine wilt disease (*bursaphelenchus xylophilus*) using remotely sensed data and gIS. Proceedings of the ACRS 2001—22nd Asian Conference on Remote Sensing.

[58] Zhou X.H., Bian G.B., Xie X.L., Hou Z.G., Li R.Q., Zhou Y.J. (2019). Qualitative and quantitative assessment of technical skills in percutaneous coronary intervention: In vivo porcine studies. IEEE Trans. Biomed. Eng..

[59] Eilertson K.E., Fricks J., Ferrari M.J. (2019). Estimation and prediction for a mechanistic model of measles transmission using particle filtering and maximum likelihood estimation. Stat. Med..

[60] Huang H.H., Ma X.H., Huang H.Y., Zhou Y.F., Zhang W., Huang Y.H. (2018). A preliminary study on monitoring of dead pine trees caused by pine wilt disease with fixed-wing unmanned aerial vehicle. J. Environ. Entomol..

[61] Li W., Shen S., He P., Hao D., Fang Y., Tao L., Zhang S. (2014). A precisely positioning technique by remote sensing the dead trees in stands with inexpensive small UAV. J. For. Eng..

[62] Tao H., Li C., Zhou J., Huai H., Jiang L., Li F. (2019). Recognition of red-attack pine trees from UAV imagery based on the HSV threshold method. J. Nanjing For. Univ. Nat. Sci. Ed..

[63] Naik S.K., Murthy C.A. (2003). Hue-preserving color image enhancement without gamut problem. IEEE Trans. Image Process. Publ. IEEE Signal Process. Soc..

[64] Takenaka Y., Katoh M., Deng S., Cheung K. (2017). Detecting forests damaged by pine wilt disease at the individual tree level using airborne laser data and worldview-2/3 images over two seasons. ISPRS Int. Arch. Photogramm. Remote Sens. Spat. Inf. Sci..

[65] Du H.Q., Ge H.L., Fan W.Y., Jin W., Zhou Y.F., Li J. (2009). Study on relationships between total chlorophyll with hyperspectral features for leaves of *Pinus masopniana* forest. Spectrosc. Spectr. Anal..

[66] Zhang S., Huang J., Qin L., Li H. (2019). Ridge regression model for estimating pine wilt disease based on hyperspectral characteristics. Trans. Chin. Soc. Agric. Mach..

[67] Wang X.T. (2011). Research on Dynamic Changes of Pine Wilt Disease Based on Hyperspectral. Master’s Thesis.

[68] Ma Y., Lv Q., Zhao X.T., He B.L., Liu H.X., Zhang X.Y. (2012). Analysis of spectral characteristics of *Pinus thunbergii* inoculated with pine wood nematode. Shandong Agric. Sci..

[69] Huang M.X., Gong J.H., Li S., Zhang B., Hao Q.T. (2012). Study on pine wilt disease hyper-spectral time series and sensitive features. Remote Sens. Technol. Appl..

[70] Torresan C., Berton A., Carotenuto F., Gennaro S.F.D., Gioli B., Matese A. (2017). Forestry applications of UAVs in Europe: A review. Int. J. Remote Sens..

[71] Tang L., Shao G. (2015). Drone remote sensing for forestry research and practices. J. For. Res..

[72] Torre-Sánchez J., Granados-López F., Castro A.I.D. (2013). Configuration and specifications of an Unmanned Aerial Vehicle (UAV) for early site specific weed management. PLoS ONE.

[73] Lehmann J., Nieberding F., Prinz T., Knoth C. (2015). Analysis of unmanned aerial system-based CIR images in forestry—A new perspective to monitor pest infestation levels. Forests.

[74] Matese A., Toscano P., Gennaro S.F.D., Genensio L., Vaccari F.P., Primicerio J., Belli C., Zaldei A., Bianconi R., Gioli B. (2015). Intercomparison of UAV, aircraft and satellite remote sensing platforms for precision viticulture. Remote Sens..

[75] Nebiker S., Lack N., Abächerli M., Laderach S. (2016). Light-weight multispectral uav sensors and their capabilities for predicting grain yield and detection plantdiseases. ISPRS Int. Arch. Photogramm. Remote Sens. Spat. Inf. Sci..

[76] Severtson D., Callow N., Flower K., Neuhaus A., Olejnik M., Nansen C. (2016). Unmanned aerial vehicle canopy reflectance data detects potassium deficiency and green peach aphid susceptibility in canola. Precis. Agric..

[77] Dash J.P., Watt M.S., Pearse G.D., Heaphy M., Dungey H.S. (2017). Assessing very high resolution UAV imagery for monitoring forest health during a simulated disease outbreak. ISPRS J. Photogramm. Remote Sens..

[78] Sandino J., Pegg G., Gonzalez F., Smith G. (2018). Aerial mapping of forests affected by pathogens using UAVs, hyperspectral sensors, and artificial intelligence. Sensors.

[79] Behmann J., Mahlein A.K., Rumpf T., Romer C., Plumer L. (2015). A review of advanced machine learning methods for the detection of biotic stress in precision crop protection. Precis. Agric..

[80] Behmann J., Acebron K., Emin D., Bennertz S., Matsubara S., Thoms S., Bohnenkamp D., Kuska M.T., Jussila J., Salo H. (2018). Specim IQ: Evaluation of a new, miniaturized handheld hyperspectral camera and its application for plant phenotyping and disease detection. Sensors.

[81] Thomas S., Kuska M.T., Bohnenkamp D., Brugger A., Alisaac E., Wahabzada M. (2018). Benefits of hyperspectral imaging for plant disease detection and plant protection: A technical perspective. J. Plant Dis. Prot..

[82] Goetz A.F.H. (2009). Three decades of hyperspectral remote sensing of the Earth: A personal view. Remote Sens. Environ..

[83] Bo W., Chen C.C., Kechadi T.M., Sun L.Y. (2013). A comparative evaluation of filter-based feature selection methods for hyper-spectral band selection. Int. J. Remote Sens..

[84] Hagen N.A., Kester R.T., Gao L.S., Tkaczyk T.S. (2012). Snapshot advantage: A review of the light collection improvement for parallel high-dimensional measurement systems. Opt. Eng..

[85] Aasen H., Burkart A., Bolten A., Baerth G. (2015). Generating 3D hyperspectral information with lightweight UAV snapshot cameras for vegetation monitoring: From camera calibration to quality assurance. ISPRS J. Photogramm. Remote Sens..

[86] Mahlein A. (2016). Plant disease detection by imaging sensors-parallels and specific demands for precision agriculture and plant phenotyping. Plant Dis..

[87] Fahlgren N., Gehan M.A., Baxter I. (2015). Lights, camera, action: High-throughput plant phenotyping is ready for a close-up. Curr. Opin. Plant Biol..

[88] Barbedo J.G.A. (2019). Detection of nutrition deficiencies in plants using proximal images and machine learning: A review. Comput. Electron. Agric..

[89] Damm A., Guanter L., Verhoef W., Schlapfer D. (2015). Impact of varying irradiance on vegetation indices and chlorophyll fluorescence derived from spectroscopy data. Remote Sens. Environ..

[90] Pinto F., Damm A., Schickling A., Panigada C., Cogliati S., Muller-Linow M., Balvora A., Rascher U. (2016). Sun-induced chlorophyll fluorescence from high-resolution imaging spectroscopy data to quantify spatio-temporal patterns of photosynthetic function in crop canopies. Plant Cell Environ..

[91] Milton E.J., Schaepman M.E., Anderson K., Kneubuhler M., Fox N. (2009). Progress in field spectroscopy. Remote Sens. Environ..

[92] Vigneau N., Ecarnot M., Rabatel G., Roumet P. (2011). Potential of field hyperspectral imaging as a non destructive method to assess leaf nitrogen content in Wheat. Field Crops Res..

[93] Behmann J., Mahlein A.K., Paulus S., Kuhlmann H., Oerke E.C., Plumer L. (2015). Calibration of hyperspectral close-range pushbroom cameras for plant phenotyping. ISPRS J. Photogramm. Remote Sens..

[94] Nagasubramanian K., Jones S., Sarkar S., Singh A.K., Singh A., Ganapathysubrmanian B. (2018). Hyperspectral band selection using genetic algorithm and support vector machines for early identification of charcoal rot disease in soybean stems. Plant Methods.

[95] Thomas S., Wahabzada M., Kuska M.T., Rascher U., Mahlein A.K. (2017). Observation of plant pathogen interaction by simultaneous hyperspectral imaging reflection and transmission measurements. Funct. Plant Biol..

[96] Elvidge C.D., Keith D.M., Tuttle B.T., Baugh K. (2010). Spectral Identification of Lighting Type and Character. Sensors.

[97] Nagasubramanian K., Jones S., Singh A.K., Sarkar S., Singh A. (2019). Plant disease identification using explainable 3D deep learning on hyperspectral images. Plant Methods.

[98] Yao Z., Lei Y., He D. (2019). Early visual detection of wheat stripe rust using visible/Near-Infrared hyperspectral imaging. Sensors.

[99] Jarolmasjed S., Khot L., Sankaran S. (2018). Hyperspectral imaging and spectrometry-derived spectral features for bitter pit detection in storage apples. Sensors.

[100] Sun Y., Wei K., Liu Q., Pan L., Tu K. (2018). Classification and discrimination of different fungal diseases of three infection levels on peaches using hyperspectral reflectance imaging analysis. Sensors.

[101] Martins G.D., de Lourdes Bueno Trindade Galo M., Vieira B.S. (2017). Detecting and mapping root-knot nematode infection in coffee crop using remote sensing measurements. IEEE J. Sel. Top. Appl. Earth Obs. Remote Sens..

[102] Liu Y.L., Qiang L., He S.L., Yi S.L., Liu X.F., Zheng Y.Q., Deng L. (2015). Prediction of nitrogen and phosphorus contents in citrus leaves based on hyperspectral imaging. Int. J. Agric. Biol. Eng..

[103] Ahmadi P., Muharam F.M., Ahmad K., Mansor S., Seman L.A. (2017). Early Detection of Ganoderma Basal Stem Rot of Oil Palms Using Artificial Neural Network Spectral Analysis. Plant Dis..

[104] Arellano P., Tansey K., Balzter H., Boyd D.S. (2017). Field spectroscopy and radiative transfer modelling to assess impacts of petroleum pollution on biophysical and biochemical parameters of the Amazon rainforest. Environ. Earth Sci..

[105] Ghamisi P., Yokoya N., Li J., Liao W.Z., Liu S., Plaza J., Rasti B., Plaza A. (2018). Advances in hyperspectral image and signal processing: A comprehensive overview of the state of the art. IEEE Geosci. Remote Sens. Mag..

[106] Zhang Z.G. (2011). The Early Identification of Remote Sensing about *Bursaphelenchus xylophilus* Based on Process Model. Master’s Thesis.

[107] Dennison P.E., Halligan K.Q., Roberts D.A. (2004). A comparison of error metrics and constraints for multiple endmember spectral mixture analysis and spectral angle mapper. Remote Sens. Environ..

[108] Clark M.L., Roberts D.A., Clark D.B. (2005). Hyperspectral discrimination of tropical rain forest tree species at leaf to crown scales. Remote Sens. Environ..

[109] Lu K.K. (2016). Prediction of the pine wood nematode based on artificial neural network and hyperspectral data. Master’s Thesis.

[110] He Q. (1999). Neural Network and Its Application in IR.

[111] Kimes D.S., Nelson R.F., Manry M.T., Fung A.K. (1998). Review article: Attributes of neural networks for extracting continuous vegetation variables from optical and radar measurements. Int. J. Remote Sens..

[112] Narendra K.S., Parthasarathy K. (1990). Identification and control of dynamical systems using neural networks. IEEE Trans. Neural Netw..

[113] Guo Y., Yu L., Oerlemans A., Lao S., Wu S., Lew M.S. (2016). Deep learning for visual understanding: A review. Neurocomputing.

[114] Xu H.C., Luo Y.Q., Zhang Y.Y., Shi Y.J. (2011). Changes of Reflectance Spectra of Pine Needles in Different Stage after Being Infected by Pine Wood Nematode. Spectrosc. Spectr. Anal..

[115] Basheer I.A., Hajmeer M. (2000). Artificial neural networks: Fundamentals, computing, design, and application. J. Microbiol. Methods.

[116] Huang G.B., Lei C. (2007). Convex incremental extreme learning machine. Neurocomputing..

[117] Kumar S.P., Sriraam N., Benakop P.G., Jinaga B.C. (2010). Entropies based detection of epileptic seizures with artificial neural network classifiers. Expert Syst. Appl..

[118] Liu W.Y. (2017). Hyperspectral estimation model for physiological parameters of pine tree under *Bursaphelenchus xylophilus* stress. Master’s Thesis.

[119] Pal M., Foody G.M. (2010). Feature Selection for Classification of Hyperspectral Data by SVM. IEEE Trans. Geosci. Remote Sens..

[120] Kong W., Zhang C., Liu F., Nie P.C., He Y. (2013). Rice seed cultivar identification using near-infrared hyperspectral imaging and multivariate data analysis. Sensors.

